# Systematic assessment of square-wave jerks in progressive supranuclear palsy: a video-oculographic study

**DOI:** 10.1007/s00415-024-12617-5

**Published:** 2024-08-12

**Authors:** Alessio Facchin, Jolanda Buonocore, Marianna Crasà, Aldo Quattrone, Andrea Quattrone

**Affiliations:** 1 Neuroscience Research Center, Department of Medical and Surgical Sciences, Magna Graecia University, Viale Europa, Germaneto, 88100 Catanzaro, Italy; 2 Institute of Neurology, Department of Medical and Surgical Sciences, Magna Graecia University, Catanzaro, Italy

**Keywords:** Progressive supranuclear palsy, Square-wave jerks, Saccadic intrusions, Parkinson’s disease, Video-oculography

## Abstract

**Background:**

The presence of frequent macro-square-wave jerks (SWJs) has been recently included in the diagnostic criteria for progressive supranuclear palsy (PSP). The aim of the current video-oculographic study was to systematically assess the presence and features of SWJs during a brief fixation task in PSP, in comparison with Parkinson’s disease (PD) patients and healthy controls (HC).

**Methods:**

Thirty-eight PSP patients, 55 PD patients and 40 HC were enrolled in the study. All patients underwent a video-oculographic (VOG) examination including a 5-s fixation task, and the number, duration and amplitude of SWJs were recorded. The diagnostic performance of several SWJs parameters were then compared in distinguishing PSP from PD patients and controls.

**Results:**

PSP patients showed a higher number and amplitude of SWJs compared to PD patients and controls. At least two SWJs within the 5-s fixation task were observed in 81.6% of PSP patients, 52.7% of PD patients and 25% of HC. The SWJs amplitude was the parameter showing the highest performances in distinguishing PSP from PD (AUC: 0.78) and HC (AUC: 0.88), outperforming the SWJ number and duration. The SWJ amplitude was larger in PSP-Richardson’s syndrome than in PSP-Parkinsonism patients, while no difference was found between PSP patients with different degrees of vertical ocular motor dysfunction.

**Conclusions:**

This video-oculographic study provides robust evidence of larger SWJs number and amplitude in PSP than in PD patients, with some potential for differential diagnosis, supporting the inclusion of this ocular sign in PSP criteria.

**Supplementary Information:**

The online version contains supplementary material available at 10.1007/s00415-024-12617-5.

## Introduction

Progressive supranuclear palsy (PSP) is a neurodegenerative disorder clinically characterised by parkinsonism, postural instability, cognitive impairment, and most importantly, ocular motor dysfunction. The diagnostic criteria developed by the Movement Disorders Society (MDS) taskforce, including different subtypes of PSP, confirmed the major diagnostic role of eye movements [1]. In addition to the well-known limitation and slowness of vertical voluntary saccades evolving into gaze palsy, the presence of square-wave jerks (SWJs) has also been included into the criteria for PSP [1] despite the paucity of large studies on this point. More in detail, according to the recent PSP diagnostic criteria, the ocular motor dysfunction can be classified into three operational definitions (O1, O2 or O3), corresponding to different levels of certainty that they contribute to the PSP diagnosis [1]. The O1 level is defined as a clear limitation of the range of gaze in the vertical plane affecting both upward and downward gaze. The O2 level is defined as decreased velocity and amplitude of vertical saccades without formal recommendation regarding upward or downward gaze. The O3 level is defined as frequent macro-SWJs or eyelid opening apraxia [1]. More attention has been paid to vertical oculomotor saccadic dysfunction while only a few data exist on SWJs in PSP and other parkinsonian syndromes [2–6].

SWJs are defined as a pair of small consecutive saccades in the horizontal plane usually less than 5–6º each, in opposite directions, during fixation [7]. In the first saccade, the eye is moved away from the fixation point, whereas in the second saccade, the eye is returned to the fixation point after a short time interval (usually less than 300 ms) [4, 6]. If a video-oculographic ocular trace is observed, it is easy to recognise the square or rectangular shape of SWJs.

A few studies have focussed on reporting the SWJs in PSP [4–6, 8], showing larger and more frequent SWJs compared to healthy participants [5–8]. However, in some studies, the evaluation was only clinical without instrumental evaluation [2] or it was based on a small group of PSP patients [5–7]. In addition, the definition and classification of SWJs was not uniform across studies [2, 3, 6, 7, 9–11] and this point may explain discrepancies in the results.

The current study aims to characterise SWJs quantitatively in PSP patients, Parkinson’s disease (PD) patients, and healthy controls (HC) using video-oculography (VOG), to investigate the SWJs prevalence and to identify the best SWJ VOG parameter to differentiate PSP from PD and HC, along with its specific diagnostic performance.

## Materials and methods

### Participants

A series of 133 participants were prospectively enrolled. They include: 38 patients with PSP, 55 patients with PD and 40 HC. Patients were recruited among those referred to the Neuroscience Research Centre at the University of Catanzaro, Italy. The diagnosis of PSP was made according to the international criteria of MDS for PSP [1]. In detail, they included 24 patients with probable PSP-Richardson’s Syndrome (PSP-RS), 13 patients with probable PSP-Parkinsonism (PSP-P) and 1 patient with probable PSP with progressive gait freezing (PSP-PGF) [1]. The PD group included 55 patients who met the MDS international PD diagnostic criteria [12]. All patients underwent clinical assessment from a movement disorder specialist, a 3-T brain MRI, and a standardised VOG assessment [13–15]. In the study, 40 HC subjects were enrolled among the partners of patients; all HC were over 50 years old, did not have a history of neurological conditions, psychiatric disorders, or any other major medical conditions, and were not currently taking any neurological or psychiatric medication. Demographic and clinical characteristics of the participants are reported in Table 1. Participants signed an informed consent form before participating in the study. The study was carried out following the guidelines given in the Declaration of Helsinki.

**Table 1:** Demographic, clinical, imaging and video-oculographic characteristics of patients with progressive supranuclear palsy, patients with Parkinson’s disease and healthy control participants

Parameter	HC (*n* = 40)	PD (*n* = 55)	PSP (*n* = 38)	*p* value
Age at examination (years)	67.7 (7.3)	67.9 (7.6)	71.2 (7.7)	n.s
Sex (F/M)	23/17	23/32	15/23	n.s
Disease duration (years)	–	5.5 (4.3)	3.8 (2.3)	n.s
MDS-UPDRS-III score	–	21.9 (14.1)	44.6 (19.9)	< 0.0001
PSPRS score	–	–	38.7 (16.9)	
H–Y score	–	1.81 (0.90)	3.33 (1.25)	< 0.0001
Ocular dysfunction (O1/O2)	–	–	17/14	
Brain MRI measurements				
MRPI	–	10.5 (2.1)	17.2 (4.4)	< 0.0001
MRPI 2.0	–	1.76 (0.76)	4.13 (1.4)	< 0.0001
VOG parameters				
A x V upward gaze	5406 (1343)	4337 (1549)	1510 (1614)	< 0.0001*
A x V downward gaze	6921 (1084)	5913 (1850)	2546 (2029)	< 0.0001*
A x V vertical gaze	6140 (971)	5072 (1438)	1952 (1629)	< 0.0001*

Note: HC = Healthy controls; PD = Parkinson’s disease; PSP = progressive supranuclear palsy; MDS-UPDRS-III = Movement Disorder Society—Unified Parkinson’s Disease Rating Scale-part III (Motor Examination); PSPRS = PSP rating scale; H–Y = Hoehn and Yahr; MRPI = Magnetic Resonance Parkinsonism Index, calculated as described in Ref. [13]; MRPI 2.0 = Magnetic Resonance Parkinsonism Index version 2.0, calculated as described in Ref. [15]; AxV = amplitude multiplied by peak velocity of saccadic eye movements, as described in Ref. [14]. The AxV was expressed in degrees^2^/s. SWJs = square-wave Jerks. *p* values represent the comparison between the three or two groups considered, depending on the variable considered. Data are expressed as mean and standard deviation in parentheses. *all corrected pairwise comparisons were significant at *p* < 0.005

### Eye-tracking recording

Video-based eye tracking system [Eye Link portable duo eye tracker (SR Research Ltd, Mississauga, Ontario, Canada)] was used to evaluate the presence of SWJs. Participants were seated in front of a computer monitor (24-inch, 531 × 299 mm) with a full HD resolution of 1920 × 1080 pixels and a refresh rate of 60 Hz. The participant’s head was stabilised by a chin rest and the viewing distance was maintained at 60 cm according to the instrument procedure. A standard 5-point calibration procedure was run before the experiment. The calibration targets were presented randomly in 5 different positions on the screen. Immediately following the calibration procedure, a 5-s fixation task was administered, instructing the participants to keep their eyes on a red point of 1° of size shown in the central position on the screen. Participants were simply required to fixate the point without any other indication of time and task. Subsequently, a task to assess upward and downward saccadic performance was performed as previously described [14].

### SWJs characterisation

The characterisation of SWJs was performed based on the following three criteria: (i) the two consecutive saccades were in opposite directions, (ii) their magnitudes were approximately similar, and (iii) they were separated by a short time interval (SWJs duration) [7]. In order to exclude possible ocular flutter, we used a minimum duration threshold of 20 ms for SWJs; for the main analyses, all SWJs with an amplitude ≥ 0.25° and duration ≥ 20 ms were selected. Since in the literature there is not a standard definition of SWJs in terms of range of duration and amplitude, different criteria were alternatively applied to characterise at best the SWJs: (i) only lower limits (> 20 ms and > 0.25°); (ii) duration < 600 ms; (iii) duration < 500 ms; (iv) duration < 400 ms; (v) duration < 300 ms; (vi) amplitude > 1°; (vii) duration < 600 ms and amplitude > 1°.

**Fig. 1:**
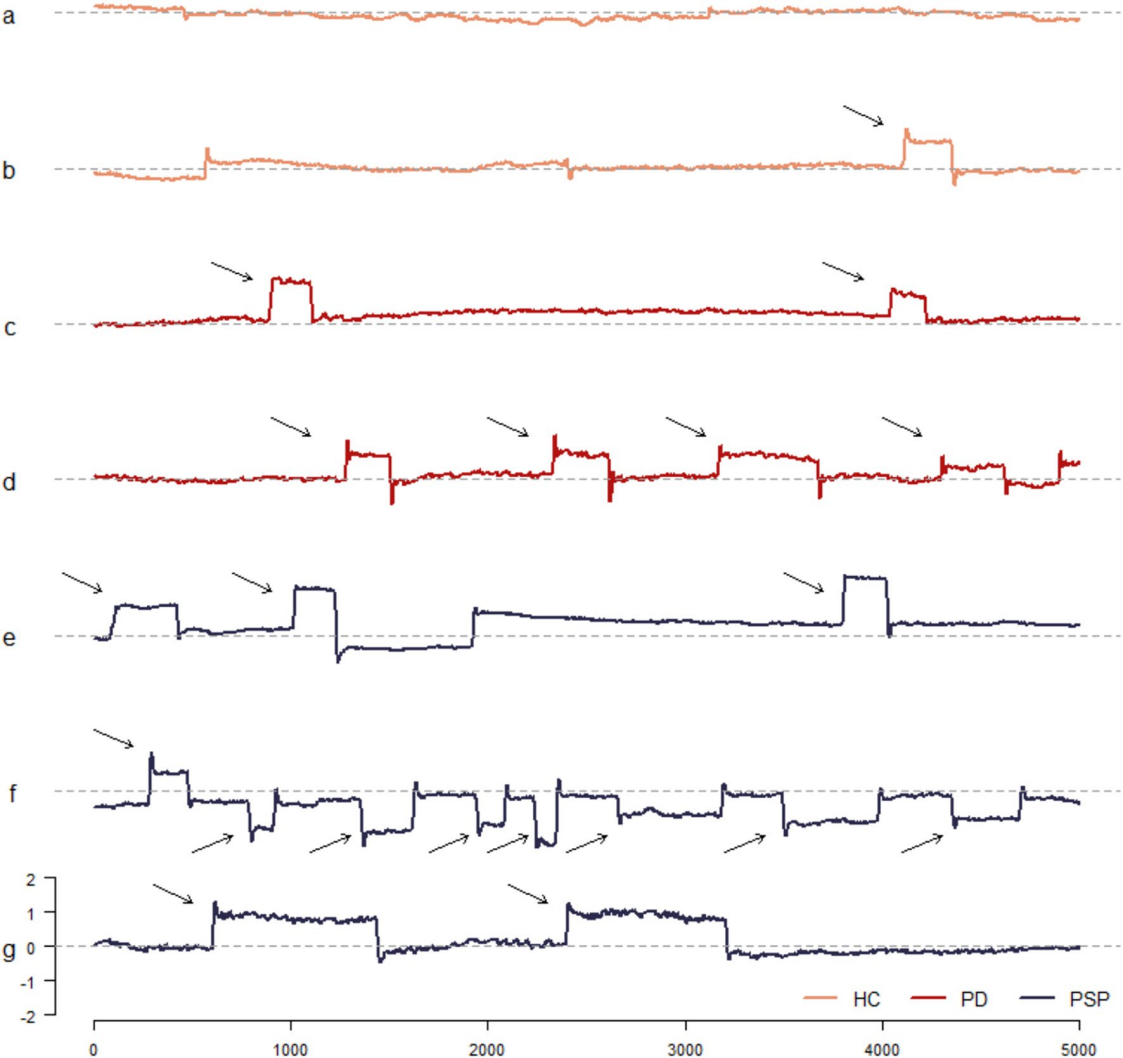
Examples of eye traces (eye position over time) during 5 s of fixation. **a**, **b** Healthy controls (HC). **c**, **d** Parkinson’s disease (PD) patients. **e**–**g** Progressive supranuclear palsy (PSP) patients. Horizontal axis represents time (ms), while vertical axis represents the eye position (º). The last trace shows the horizontal eye position and timescale for all traces. Arrows indicate SWJs

Eye tracking data during fixation were exported and subsequently analysed as follows. Eye movement position log data were exported in a raw format using EyeLink Data Viewer software (version 3.2.1 SR Research Ltd., Mississauga, Ontario, Canada). The visualisation of SWJs was performed by tracing a scatterplot image of horizontal eye position in which the X axis represents the time (0–5000 ms) and the Y axis represents the eye position (± 6° from fixation point), similarly to Fig. 1. For each participant, an image was created with a resolution of 2000 × 1000 pixels using R statistical environment and base graphic system [16]. Subsequently, three raters blinded to the clinical diagnosis performed the qualitative description of SWJs in terms of presence/absence of SWJs and number of SWJs observed within 5 s. The disagreement between evaluations was then resolved using a consensus between raters (Beaton et al., 2000). The quantitative characterisation of SWJs was performed analysing each eye tracing image with the Open Source software ImageJ (https://imagej.net/ij/ version 1.5.4 g), in terms of duration (x axis) and amplitude (y axis) using the rectangle selection tool. The results in pixels were converted to duration expressed in milliseconds and amplitude expressed in degrees. The same procedure has already been used to analyse tracing data [17, 18]. As a result, all SWJs previously defined were quantified in terms of duration and amplitude.

### Statistical methods

Demographic, clinical and VOG data were compared across groups using Kruskal–Wallis test or Wilcoxon sign Rank test, as appropriate. The agreement among the three raters in terms of the presence/absence of SWJs was performed with Fleiss K [19], while the agreement on the number of SWJs detected in each patient was performed using intra class correlation ICC (1,1) [20, 21]. Diagnostic classification metrics were calculated for different SWJs parameters in the comparison between PSP and HC groups and between PSP and PD groups in terms of sensitivity, specificity, accuracy, and area under the curve (AUC). The following VOG parameters of SWJs were tested: (i) number of SWJs, (ii) mean duration, (iii) mean amplitude, (iv) mean duration x mean amplitude and (v) total SWJs amplitude (sum of all SWJs amplitudes). Diagnostic cutoffs were calculated using the criterion aimed to maximise sensitivity and specificity [22]. Subsequently, different criteria aimed to define SWJs were compared in terms of AUC in distinguishing PSP from PD and HC. Comparisons of AUC were performed using the DeLong Test. Finally, correlation analyses were performed between the SWJs parameter and a series of demographic, neurological and clinical variables using Pearson or Spearman correlation coefficient, correcting for false discovery rate. Analysis and graphical representation were performed using R statistical environment and specific packages [16].

## Results

### Clinical evaluation

The demographic, clinical, imaging and video-oculographic data of PSP patients, PD patients and HC are shown in Table 1.

No differences were found in age and sex among the three groups. PSP patients had higher disease severity than PD patients despite similar disease duration. PSP patients had higher MRPI and MRPI 2.0 values compared to PD patients, calculated as previously described. Video-oculographic assessment revealed upward or downward dysfunction in 85.2% of PSP patients and none of HC; 23.7% of PSP has upward dysfunction only, 5.3% has downward dysfunction only and 55.3% both. A few PD patients (10.9%) had mild upward dysfunction while no PD patient had downward VOG dysfunction.

## SWJs assessment

**Fig. 2:**
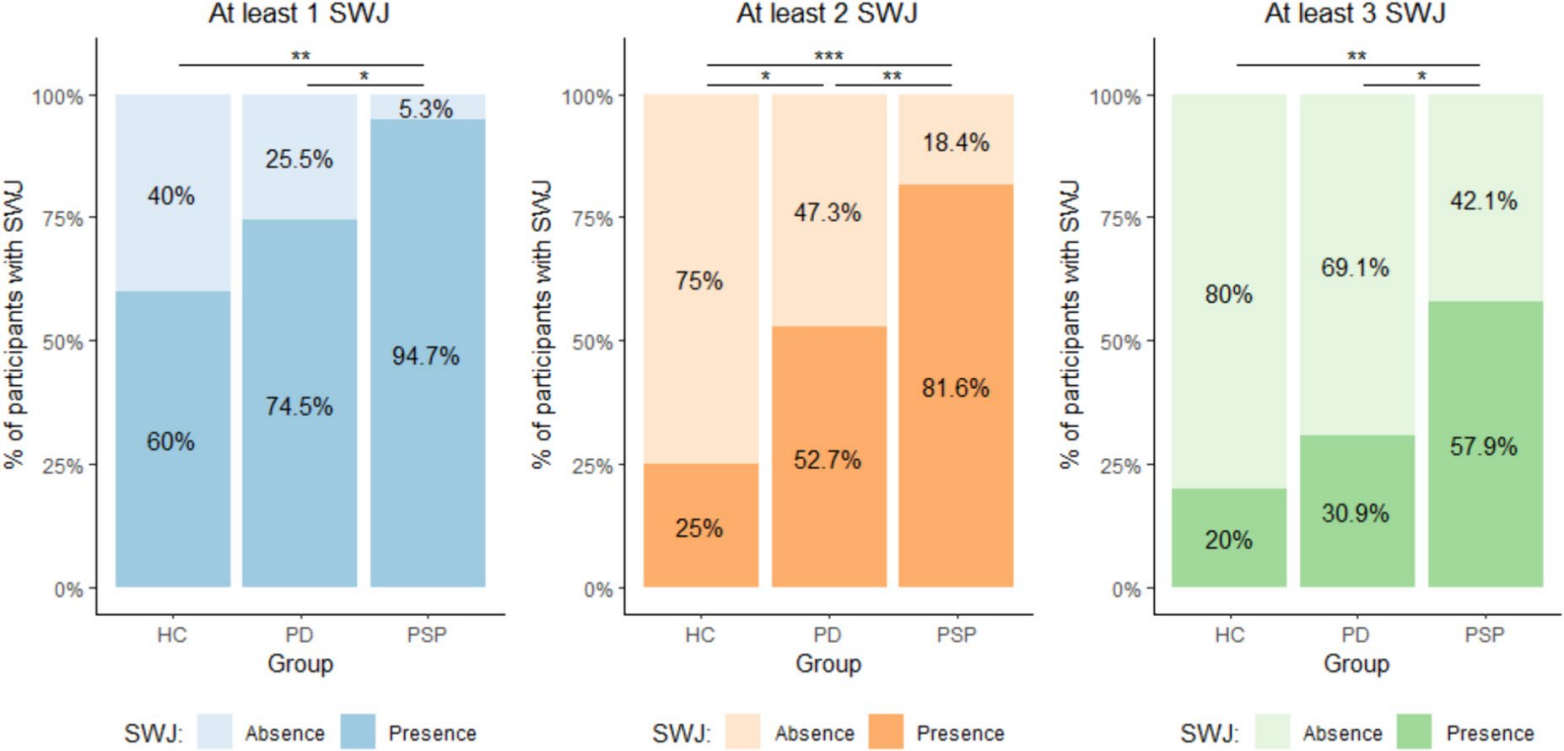
SWJ = square-wave jerks. Graphs showing the percentage of patients with or without SWJs according to the criteria of at least 1 SWJs (left), at least 2 SWJs (centre), and at least 3 SWJs (right) in 5 s. * = *p* < 0.05; ** = *p* < 0.01; *** = *p* < 0.001

Three independent raters assessed the presence and number of SWJs on eye traces. Overall, an almost perfect agreement, K = 0.84, *p* < 0.0001 was reached, with an excellent reliability, ICC = 0.96 of these evaluations. Overall, the amplitude of single SWJs ranged between 0.26° and 5.65°. The percentage of subjects showing at least one, two or three SWJs within 5 s, is shown in Fig. 2. At least two SWJs within this timeframe were observed in above 80% of PSP patients, around 50% of PD patients and only in 25% of HC.

In comparing the SWJs characteristics, PSP patients showed a higher number of SWJs, larger SWJs amplitude, larger SWJs amplitude x duration and larger total SWJs amplitude compared to PD and HC (all *p* values < 0.01; Table 2).

**Table 2:** SWJs video-oculographic features in patients and control subjects

Parameter	HC (*n* = 40)	PD (*n* = 55)	PSP (*n* = 38)	*p* value
SWJs number	1.25 (1.50)	1.89 (1.66)	3.18 (1.94)	< 0.0001 a,b,c
SWJs mean amplitude (deg.)	0.36 (0.35)	0.55 (0.42)	1.12 (0.82)	< 0.0001 a,b,c
SWJs mean duration (msec.)	191 (195)	254 (202)	307 (163)	< 0.05 c
Amplitude x duration (deg. x msec.)	112 (124)	182 (164)	362 (300)	< 0.0001 a,b,c
Total SWJs amplitude (deg.)	0.77 (1.05)	1.42 (1.46)	3.86 (4.33)	< 0.0001 a,b,c
Number of blinks	0.36 (0.85)	0.21 (0.62)	0.29 (0.65)	n.s

Note: SWJ = square-wave jerks; HC = healthy controls; PD = Parkinson’s disease; PSP = progressive supranuclear palsy; data are reported as mean (standard deviation). deg. = degrees. msec = milliseconds. Total SWJ amplitude was the sum of the amplitudes of each SWJs performed within the 5-s fixation task (also corresponding to the mean SWJ amplitude multiplied by the number of SWJ). *p* value = result of Kruskal–Wallis test. n.s. = not significant. Holm-corrected pairwise comparisons significant at *p* < 0.05 or lower were reported for the comparisons: a = PD vs HC; b = PD vs PSP; c = HC vs PSP

Among PSP patients, the total SWJs amplitude was significantly larger in PSP-RS (4.78°) than in PSP-P patients (2.13°; W = 79, p < 0.05) despite a similar SWJs number and duration, while no difference was found between PSP patients with different degrees of ocular motor dysfunction (O1 and O2). In both PSP and PD groups taken separately, no significant correlation was found between SWJs parameters and demographic, clinical, VoG (saccadic amplitude multiplied by peak velocity) and imaging data.

### SWJs classification performance

The classification performances of different SWJ parameters (number, amplitude, duration and combined indexes) were compared, as shown in Table 3. The best parameter to differentiate PSP from PD and HC was the SWJs amplitude (mean or total), which reported an AUC = 0.88 in differentiating PSP from HC and AUC = 0.77–0.78 in differentiating PSP from PD patients (Table 3), outperforming SWJs number and duration. The optimal cutoffs to maximise sensitivity and specificity are reported in Table 3.

**Table 3:** Classification parameters of different SWJ parameters to differentiate PSP from HC and PD patients

Parameter	Number of SWJs		Mean duration		Mean amplitude		Mean duration x mean amplitude		Total SWJ amplitude	
Groups	PSP vs HC	PSP vs PD	PSP vs HC	PSP vs PD	PSP vs HC	PSP vs PD	PSP vs HC	PSP vs PD	PSP vs HC	PSP vs PD
Cutoff	2	3	251	262	0.64	0.86	182.9	238.7	1.37	1.88
Sensitivity	0.82	0.61	0.63	0.53	0.82	0.71	0.82	0.66	0.87	0.76
Specificity	0.73	0.69	0.65	0.49	0.83	0.78	0.78	0.67	0.80	0.73
Accuracy	0.77	0.66	0.64	0.51	0.82	0.75	0.79	0.67	0.83	0.74
AUC	0.80	0.69	0.68	0.57	0.88	0.77	0.84	0.73	0.88	0.78

Note: SWJ = square-wave jerks; HC = healthy controls; PD = Parkinson’s disease; PSP = progressive supranuclear palsy; AUC = area under the curve

### SWJs definition criteria

SWJs were defined as involuntary saccadic intrusions during fixation with typical duration of 200–300 ms [1]; however, no specific criteria on amplitude and maximum duration have been described so far to define SWJs unambiguously. Here, we first analysed all visible SWJs with amplitude > 0.25° (range: 0.25°–5.13°) and duration > 20 ms (range: 117 ms–842 ms.), as discussed above. Subsequently, we repeated the SWJs assessment multiple times by varying the SWJs inclusion criteria, aiming to best identify SWJs typically observed in PSP patients and not in PD and HC. All classification parameters are reported in Supplementary Tables 1 and 2. The best classification performance in ROC analysis in differentiating PSP from HC and PD was obtained using all visible SWJs, with no classification improvement after restricting the analysis to SWJs of short duration (200–300 ms) or to large SWJs > 1° (Supplementary Table 1).

## Discussion

This study aimed at characterising the SWJs in PSP, PD and healthy controls in a quantitative way using VOG, to investigate the SWJs prevalence and to identify the best SWJs VOG parameter that differentiates PSP from PD and HC. The main results of this study showed robust evidence of larger SWJs number and amplitude in PSP compared to PD patients and healthy controls.

SWJs are usually described as typical of PSP patients and consequently they are included in the PSP diagnostic criteria developed by the MDS. Taking into account the presence of at least 1 SWJs, the large part of PSP (~ 95%) showed SWJs, and this was in line with the ~ 88% prevalence found by Rascol in a small group of 12 PSP patients [5]. Considering the number of SWJs in a definite amount of time, in PSP patients, we observed a mean of 3.2 SWJs within 5 s. This is in line with other reports of 4.0 [7], 3.8 [2] and 2.8 SWJs [6] in the same time interval. Observing the characteristics of SWJs in a quantitative way measured using VOG, PSP patients had a larger SWJs amplitude than HC, in agreement with previous evidence [7]. The mean amplitude of SWJs was not related to the number of SWJs, which is consistent with other studies [9].

It is well recognised that PSP is a heterogeneous condition [23], and PSP-RS represents a more severe condition and exhibits serious ocular motor dysfunction. In our study, no differences were found between PSP subtypes (PSP-RS vs PSP-P) in the number of SWJs performed, in line with other reports [2], but there was a significant difference in total SWJs amplitude, with PSP-RS patients showing larger SWJs than PSP-P patients. On the contrary, no differences in SWJs were found between patients with different degrees of oculomotor dysfunction (O1 and O2) and no correlation was found between SWJs parameters and vertical eye movement VOG performance. Taken together, these results demonstrate that SWJ are evident not only in patients with gaze palsy, but also in those with mild vertical ocular dysfunction (O2) and suggest different anatomical substrates for SWJs and vertical oculomotor dysfunction. Indeed, this latter seems to be related to hypometabolism of bilateral anterior cingulate gyrus and right lingual gyrus [24], reduced metabolism in superior colliculi [25] and to the involvement of rostral interstitial nucleus of the medial longitudinal fasciculus [26–28]. Conversely, supratentorial cortical structures located primarily in the temporal lobe, together with the anatomical connections of the temporal lobe to the superior colliculus and the cerebellum seem to play an important role in the generation of abnormally high SWJs rates in PSP patients [6].

Since SWJs are included in the PSP diagnostic criteria, it is interesting not only the comparison between PSP and HC but most importantly, the comparison between PSP and PD to evaluate the real usefulness of this ocular sign in the differential diagnosis of parkinsonian syndromes. In PD patients, we observed a frequency of 1.9 SWJs within 5 s, and this is in line with a recent study [29], and slightly higher than others [2, 6]. SWJs data did not seem to be related to age or disease duration in PD patients. Among the different SWJ parameters, the total and mean amplitude of SWJs produced the best results in distinguishing PSP from PD (AUC = 0.78) and from HC (AUC = 0.88), differentiating PSP from PD patients and HC, with some overlap at individual level. This result is in line with the inclusion of this ocular sign in MDS-PSP criteria though with lower certainty degree for PSP pathology compared with vertical ocular slowness or gaze palsy. This study also demonstrated a superiority of amplitude over SWJs number in supporting PSP diagnosis, highlighting the usefulness of VOG technique which allows an accurate amplitude quantification. The SWJs number within a brief time interval, on the other hand, may be more easily assessed at the bedside examination than SWJ amplitude, making this parameter potentially useful for clinicians in the absence of eye movement recording devices.

Over a multiplicity of studies assessing SWJs in patients with various neurological and paediatric disorders [4, 11, 30–34], no consensus has been reached regarding the definition of SWJs in terms of amplitude and duration [2, 4–7, 9, 35–37]. In this study, we thus performed a formal comparison of different SWJs inclusion criteria aiming at identifying the SWJs definition criteria most useful for differentiating PSP from PD patients and HC. We obtained the best performance by including all SWJs with at least 0.25° of amplitude and 20 ms of duration [7], rather than restricting the analysis to SWJs of shorter duration or amplitude larger than 1º, as performed in previous studies.

This study has several strengths. First, all study participants underwent a standardised VOG assessment and the presence of SWJs was assessed by three expert raters. Second, we used a comprehensive approach to compare different SWJs parameters and SWJs definition criteria, providing robust evidence to improve future research on this topic.

As with any study, there are limitations. The patients in this study did not undergo a pathological examination. The clinical diagnosis, however, was made in accordance with international diagnostic criteria by movement disorder specialists. SWJs were detected on the ocular trace in a semi-quantitative manner, as done previously in most studies. However, this was performed by three expert raters with good inter-rater agreement. Finally, our fixation task was quite short; SWJ typically do not occur at regular intervals and longer recordings may allow a more comprehensive assessment of SWJ presence and number. On the other hand, however, a brief fixation task of 5 s may increase the compliance to the test which can be reduced in patients with neurodegenerative disease, and may provide results more comparable with the clinical assessment that typically lasts a few seconds.

In summary, this study demonstrates the importance of VOG assessment of SWJs to support PSP diagnosis, supporting the inclusion of this ocular sign in PSP diagnostic criteria, and identified the SWJs amplitude as the most powerful feature for patient classification. In addition, this study provides useful guidance on SWJs inclusion criteria in video-oculographic studies to improve future research in the field.

## Supplementary Information

Below is the link to the electronic supplementary material.

Supplementary file1 (DOCX 22 KB)

## Data Availability

Anonymised data that support the results of this study are available from the corresponding author upon reasonable request.
