# Supplementary material for: Systematic assessment of square-wave jerks in progressive supranuclear palsy: a video-oculographic study
Source: J Neurol. 2024 Aug 12;271(10):6639–46. doi: 10.1007/s00415-024-12617-5 (PMC11447104; doi:10.1007/s00415-024-12617-5)
Supplement: Supplementary file 1 — Supplementary file1 (DOCX 22 KB) [file 415_2024_12617_MOESM1_ESM.docx]

**Supplementary materials**

**Table S1.** Classification performances obtained using different parameters and different definition criteria of SWJs.

|  |  | Number of SWJs | Mean SWJs amplitude | Total SWJs amplitude |
| --- | --- | --- | --- | --- |
| All SWJs | PSP vs HC | 0.80 | 0.88 | 0.88 |
|  | PSP vs PD | 0.69 | 0.77 | 0.78 |
| SWJs <600ms | PSP vs HC | 0.76 | 0.84 | 0.84 |
|  | PSP vs PD | 0.69 | 0.77 | 0.75 |
| SWJs <500ms | PSP vs HC | 0.77 | 0.84 | 0.84 |
|  | PSP vs PD | 0.69 | 0.76 | 0.76 |
| SWJs <400ms | PSP vs HC | 0.76 | 0.82 | 0.82 |
|  | PSP vs PD | 0.68 | 0.74 | 0.74 |
| SWJs <300ms | PSP vs HC | 0.73 | 0.80 | 0.79 |
|  | PSP vs PD | 0.66 | 0.73 | 0.71 |
| SWJs >1° | PSP vs HC | 0.81 | 0.80 | 0.82 |
|  | PSP vs PD | 0.76 | 0.77 | 0.77 |
| SWJs <600 ms & >1° | PSP vs HC | 0.80 | 0.78 | 0.79 |
|  | PSP vs PD | 0.74 | 0.76 | 0.76 |

Note: SWJ = square wave jerks; HC = Healthy controls; PD = Parkinson’s disease; PSP = progressive supranuclear palsy; Data are area under the curve (AUC) values.

**Table S2.** number and percentage of patients with or without SWJs according to the different SWJs definition criteria.

| Criteria | Presence of | HC | PD | PSP |
| --- | --- | --- | --- | --- |
| All SWJs | at least 1 SWJs | 24 (60%) | 41 (74.5%) | 36 (94.7%) |
|  | at least 2 SWJs | 10 (25%) | 29 (52.7%) | 31 (81.6%) |
|  | at least 3 SWJs | 8 (20%) | 17 (30.9%) | 22 (57.9%) |
| SWJs <600ms | at least 1 SWJs | 23 (57.5%) | 39 (70.9%) | 31 (81.6%) |
|  | at least 2 SWJs | 10 (25%) | 25 (45.5%) | 26 (68.4%) |
|  | at least 3 SWJs | 8 (20%) | 15 (27.3%) | 17 (44.7%) |
| SWJs <500ms | at least 1 SWJs | 21 (52.5%) | 38 (69.1%) | 31 (81.6%) |
|  | at least 2 SWJs | 10 (25%) | 22 (40%) | 24 (63.2%) |
|  | at least 3 SWJs | 7 (17.5%) | 13 (23.6%) | 17 (44.7%) |
| SWJs <400ms | at least 1 SWJs | 19 (47.5%) | 37 (67.3%) | 30 (78.9%) |
|  | at least 2 SWJs | 9 (22.5%) | 20 (36.3%) | 24 (63.2%) |
|  | at least 3 SWJs | 6 (15%) | 11 (20.0%) | 16 (42.1%) |
| SWJs <300ms | at least 1 SWJs | 17 (42.5) | 31 (56.3%) | 30 (78.9%) |
|  | at least 2 SWJs | 7 (17.5%) | 15 (27.3%) | 20 (52.6%) |
|  | at least 3 SWJs | 3 (7.5%) | 6 (10.9%) | 11 (28.9%) |
| SWJs >1° | at least 1 SWJs | 6 (15%) | 16 (29.1%) | 28 (73.7%) |
|  | at least 2 SWJs | 1 (2.5%) | 3 (5.5%) | 16 (42.1%) |
|  | at least 3 SWJs | 0 (0%) | 2 (3.6%) | 8 (21.1%) |
| SWJs <600ms & >1° | at least 1 SWJs | 6 (15%) | 15 (27.3%) | 26 (68.4%) |
|  | at least 2 SWJs | 1 (2.5%) | 3 (5.5%) | 16 (42.1%) |
|  | at least 3 SWJs | 0 (0%) | 2 (3.6%) | 7 (18.4%) |

Note: SWJ = square wave jerks; HC = Healthy controls; PD = Parkinson’s disease; PSP = progressive supranuclear palsy; Data are number and % of patients in each group.
